# Food labelling in India: a scoping review of consumer engagement, comprehension, and purchase behaviour

**DOI:** 10.1080/16549716.2025.2574132

**Published:** 2025-11-11

**Authors:** Maneesha Pahlani, Kandarp Narendra Talati, Sandra Lopez-Arana, Prakash Narayanan

**Affiliations:** a Department of Global Public Health Policy and Governance, Prasanna School of Public Health, Manipal Academy of Higher Education, Manipal, India; b School of Nutrition and Dietetics, Faculty of Medicine, Finis Terrae University, Providencia, Chile

**Keywords:** nutrition literacy, front-of-package labelling, food policy, health promotion, Sustainable Development Goals

## Abstract

Amid rising packaged food consumption in India and its associated health risks – including obesity and non-communicable diseases – this scoping review synthesised India-specific evidence on consumer awareness, comprehension, and behavioural responses to food labels on pre-packaged products. Following the Preferred Reporting Items for Systematic Reviews and Meta-Analyses extension for Scoping Reviews checklist and Arksey and O’Malley framework, five databases (PubMed, Scopus, Embase, Web of Science, and CINAHL) and Google Scholar were searched for peer-reviewed primary studies conducted in India between 2014 and 2024. Studies were included if they assessed food label literacy, interpretation, or use in purchase behaviour in Indian settings. Thirty-two studies were included, covering diverse populations and geographic settings. Bibliometric synthesis showed a predominance of cross-sectional knowledge, attitudes, and practices studies, with limited experimental or multidisciplinary research. Findings were organised into three analytical themes: a) determinants of food label literacy, including socio-demographic and cognitive factors; b) consumer perceptions of label components, their placement on packaging, visual appeal, and cognitive utility; and c) behavioural implications of label engagement and its perceived influence on purchase intentions. Evidence from this review highlights persistent gaps between label awareness, engagement, and purchase intentions. To inform policy and practice – and to advance Sustainable Development Goal 3 (Target 3.4 – reducing premature mortality from non-communicable diseases) & Goal 12 (Target 12.8 – promoting awareness and information access) – future research should prioritise experimental and implementation-focused designs tailored to India’s demographic, cultural, and market heterogeneity.

## Paper Context

- **Main findings:** Indian consumers prioritise expiry date and price over nutritional information, and given the reliance on self-reported behaviours, further research is needed to understand how socio-demographiic differences, brand familiarity, and affordability interact with food label engagement to shape actual purchase decisions.
- **Added knowledge:** India-specific evidence in this review highlights the need for an integrated approach to food label research, discussed through real-world examples of policy and programme convergence to enhance label literacy and foster informed consumer choices.
- **Global health impact for policy and action:** Emerging technologies, strategic policy convergence together with inter-sectoral collaborations to integrate food labels with nutrition, education, and sustainability initiatives—can bridge the global gap between consumer awareness, intention, and actual purchase decisions as well as to enable food labels to play a more influential role in supporting healthier, better informed, and sustainable choices worldwide.

## Background

Enhancing food label literacy has emerged as a critical priority in public health, particularly as the consumption of processed and packaged foods increases, contributing to the rising prevalence of non-communicable diseases (NCDs) [1–3]. Ultra-processed high-fat, sugar, and salt (HFSS) foods are becoming more affordable and accessible. According to the Indian Council of Medical Research – National Institute of Nutrition (ICMR – NIN), unhealthy diets are responsible for approximately 56.4% of India’s total disease burden [3]. The market size of the packaged food sector in India is expected to grow from US$2.8 billion in 2023 to US$6.4 billion by 2029, with snacks and sweets accounting for approximately one-third of the market share [4]. Aggressive marketing of HFSS foods – primarily through digital and social media – significantly influences dietary preferences, particularly among children and young adults, thereby contributing to long-term health risks [3]. Tackling the double burden of malnutrition in the Indian context, where undernutrition persists alongside rising rates of overweight, obesity, and metabolic conditions, demands urgent public health attention [5,6]. Building food label literacy – enabling consumers to access, understand, and use label information – is a key strategy for improving public health outcomes [7,8].

Food labels serve as a critical interface between consumers and food products, providing information on nutritional content, ingredients, and safety [9,10]. Globally, front-of-pack labels (FoPL), nutrient declarations, and health logos are promoted to encourage healthier purchasing behaviour [11,12]. In India, the Food Safety and Standards Authority of India (FSSAI) has mandated comprehensive labelling norms for pre-packaged foods under the Food Safety and Standards (Labelling and Display) Regulations, 2020. These regulations specify mandatory front-of-pack declarations, including the name of the food and the vegetarian or non-vegetarian logo. In addition, every package must display key information, including a list of ingredients, nutritional details, allergens, food additives, date marking, batch number, license number, and instructions for use – without a specific requirement about placement [13].

Nonetheless, much of the global evidence on food labelling – particularly on front-of-pack labelling formats, consumer engagement, and their influence on purchasing behaviour – is concentrated in high-income countries with well-regulated retail and nutrition environments, making it less directly transferable to low- and middle-income countries such as India. Factors such as varying nutrition literacy levels, language barriers, differing retail ecosystems, and cultural attitudes towards packaged food may mediate how consumers engage with label content. The growing autonomy of young adults – especially university students – regarding food choices further underscores the importance of how nutrition literacy influences food label awareness and purchasing decisions [14,15]. Moreover, India’s National Education Policy (NEP) 2020 emphasises multidisciplinary education and research to solve real-world challenges [16]. In this light, food label engagement offers a fertile ground for collaboration across public health, nutrition science, behavioural research, education, marketing, mass media communications, and policy domains.

Against this backdrop, this scoping review aims to systematically map India-specific evidence on consumer engagement (awareness of and attention to label components), comprehension (understanding of label information), and behavioural response (application of label information while making purchase decisions) to food labelling on pre-packaged products – attuned to the country’s demographic and retail diversity.

## Methods

### Search strategy

A comprehensive search was conducted across five major bibliographic databases – PubMed, Scopus, Embase, Web of Science, and CINAHL – along with Google Scholar (as an additional source), to identify relevant published literature between 2014 and 2024. The search strategies were completed using keywords ‘food labelling’, ‘food label’, ‘nutrition* label’, ‘food label use’, ‘purchasing behaviour’, ‘purchase intention’, ‘buying behaviour’, ‘buying intention’, ‘food choice*’, ‘India’. Boolean operators AND/OR were used to combine the search terms appropriately, and keywords were adapted for each database. The detailed search strategy is provided in Supplementary Table S1. The database searches were conducted on 17 February 2025 for PubMed, Scopus, CINAHL, and Embase, and on 20 February 2025 for Web of Science and Google Scholar. The review was conducted in accordance with the Preferred Reporting Items for Systematic Reviews and Meta-Analyses extension for Scoping Reviews (PRISMA-ScR) guidelines [17].

### Eligibility criteria

Studies were eligible for inclusion if they were primary research articles published in peer-reviewed journals, including qualitative, quantitative, or mixed-method designs; were conducted in India between 2014 and 2024; published in English; and assessed consumer engagement, literacy, or behaviour related to food labels on prepackaged foods. Studies were excluded if they were non-primary studies (e.g. reviews, meta-analyses, commentaries, editorials, book chapters, reports, or correspondence); focused exclusively on disease-specific or medically diagnosed populations; addressed non-prepackaged foods (e.g. menu labels, cafeteria meals, fresh produce); or if the full article was not accessible.

### Study selection

**Figure 1.**
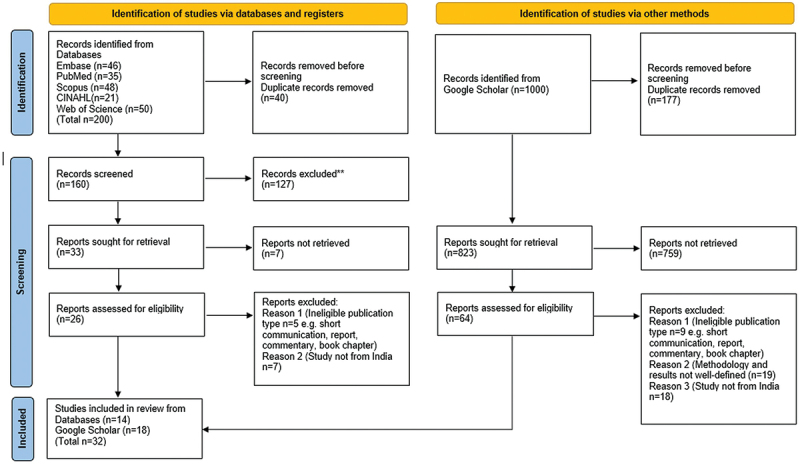
PRISMA-ScR flow diagram illustrating the study selection process.

The PRISMA flow diagram (Figure 1) presents an overview of the study selection process and was prepared in accordance with the PRISMA 2020 flow diagram for new systematic reviews, which includes searches of databases, registers, and other sources [18]. Given the limitations of Google Scholar’s indexing and ranking algorithms, we screened the first 1,000 results to ensure a broader capture of India-specific literature not indexed in conventional academic databases. A total of 1,200 articles were initially identified across multiple databases. Based on title and abstract screening after removing 217 duplicates and excluding 864 records using the Rayyan tool, 119 full-text articles were assessed for eligibility. Of these, inclusion criteria were not met in 87, resulting in 32 studies being included in the final thematic synthesis.

### Data extraction and synthesis

The review team developed a structured data extraction form to systematically capture relevant information from each included study. Extracted fields included: author(s), publication year, source/database, study location, objectives, methodological approach (quantitative, qualitative, or mixed methods), participant characteristics, and sample size, key findings related to food label awareness, comprehension, use, and influence on purchasing behaviour. Information on disciplinary affiliations, institutional collaborations, and funding sources was recorded, where available, to support an assessment of research networks. Data on outcomes such as consumer awareness of FSSAI regulations, KAP scores, and similar measures were not extracted, as they were beyond the scope of this review.

Two independent reviewers (MP and KT) performed the title and abstract screening, followed by full-text review. Discrepancies were resolved through discussion, and when consensus could not be reached, a third reviewer (PN) was consulted to adjudicate. Two reviewers independently performed data extraction using a pre-designed template. Extracted data was compared and cross-verified for consistency, and disagreements were resolved through mutual consensus.

A thematic synthesis was undertaken to collate and summarise findings on food label engagement, comprehension, and purchase behaviour in the Indian context. The synthesis derived conceptual grounding from the Scoping Review Framework of Arksey and O’Malley, with emphasis on systematically mapping conceptual domains, methodological approaches, and knowledge gaps to inform evidence-based research and policy decisions [19]. Studies were first mapped according to methodological design, study population, and disciplinary context. Key findings were organised into three analytical themes to report the findings.

## Results

**Figure 2.**
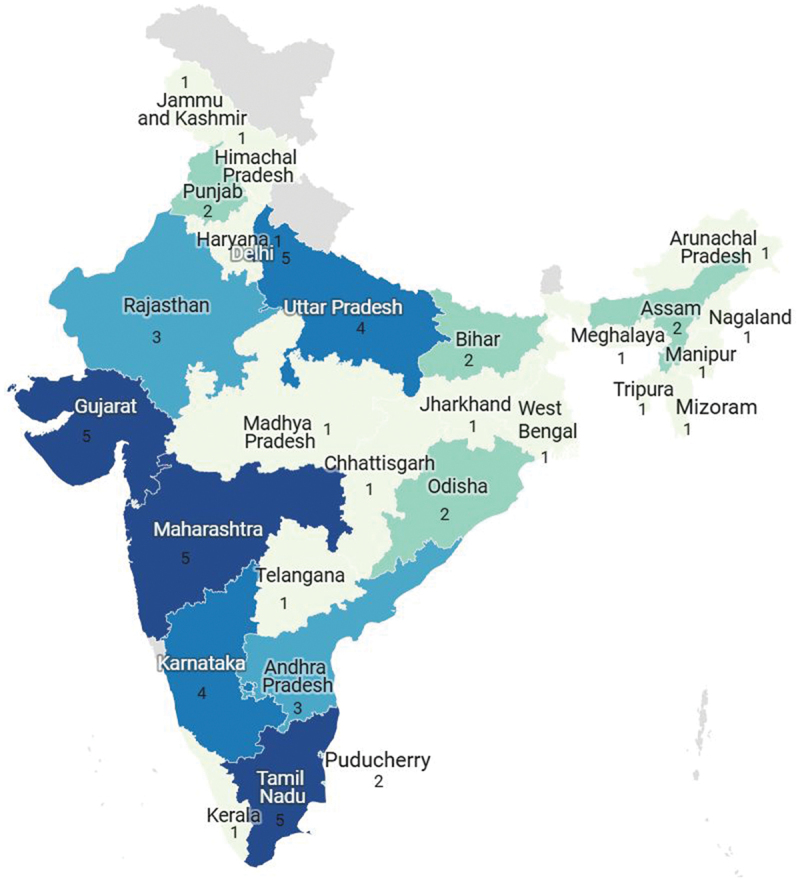
Geographical distribution of included studies (2014-2024) across Indian states and union territories.

*Note*: Including four multi-centre studies that covered more than one state/UT.

The 32 studies, including four multi-centre studies, spanned multiple Indian states and involved participants from diverse rural and urban contexts. Seven publications were identified between 2015–2020, all indexed in Google Scholar. The number increased to 25 publications between 2021–2024, including 11 from Google Scholar. Notably, 17 publications were from 2023–2024, underscoring growing scholarly engagement aligned with India’s policy focus on FoPL guidelines. Quantitative designs (*n* = 29) predominated, with fewer studies employing qualitative (*n* = 1) or mixed-method (*n* = 2) approaches. Many studies focused on knowledge, attitudes, and practices (KAP), with only five specifically examining FoPL, and just four adopting experimental designs to assess consumer responses objectively. Sample sizes ranged from 44 to over 20,000 participants, covering adolescents, young adults, and adults (Supplementary Table S2). As illustrated in Figure 2, consumer representation was higher in Tamil Nadu, Maharashtra, Gujarat, and Delhi (*n* = 5 each), followed by Karnataka and Uttar Pradesh (*n* = 4 each), and Rajasthan and Andhra Pradesh (*n* = 3 each). Four studies didn’t report study locations. Data collection settings varied equally – from schools and colleges to supermarkets, online surveys, and controlled experimental environments such as digital eye-tracking laboratories to assess label engagement patterns.

Eighteen of the 32 studies were sourced from Google Scholar, reflecting the platform’s utility in surfacing context-specific literature not always indexed in conventional academic databases. Nearly half of the studies (*n* = 16) were conducted by medical colleges; others emerged from institutions focused on nutrition science, food technology, agri-business, marketing, or public health. Single-department authorship was common (*n* = 21), most frequently from Community Medicine (*n* = 13). Eleven studies demonstrated interdepartmental collaboration, drawing on complementary expertise from Sociology, Nutrition Science, Food Policy, Marketing, and Agricultural Economics. Notably, none of the included studies reported industry funding or collaboration. Nine studies, including four short-term studentships for medical undergraduate and a research fellowship, acknowledged financial or conceptual support from national/international agencies and institutional intramural schemes – for instance, FSSAI, ICMR, ICMR-NIN, University Grants Commission – India, Jawaharlal Institute of Postgraduate Medical Education and Research (JIPMER), the World Health Organization Country Office for India, Resolve to Save Lives and Bloomberg Philanthropies [20–28]. Despite these instances, broader cross-sectoral collaboration remained limited.

Thematic synthesis of the included studies, presented below, revealed three dominant patterns: (a) determinants of food label literacy; (b) consumers’ perceptions of label utility and design features; and (c) the influence of food label engagement on purchase intentions.

### Determinants of food label literacy

Socio-demographic characteristics showed varied associations with food label awareness, use, and comprehension. Multiple studies found that gender, age, education level, parental education, income, marital status, dietary preferences, presence of co-morbidities, and residential locality shaped consumer engagement with food labels [25,26,29–37]. For example, women, younger adults, and individuals with higher education were more likely to report reading labels, most frequently expiry dates. Income and occupation were also reported to influence food label use in some studies [22,23,32,36,38,39]. However, these associations were not uniform across all settings. Some studies found no significant relationship between label literacy and socio-demographic factors or household health conditions, including non-communicable diseases [27,40,41]. Another study reported education and gender as significant predictors of food label engagement, but not age or occupation [42]. Gender, in particular, emerged as a consistent determinant across studies, with female participants more frequently engaging with diverse label components.

However, awareness did not always translate into food label comprehension or effective use in purchase decisions. Few studies reported that although participants recognised nutrition terms or FOP symbols, they often could not interpret them correctly [26,43]. Respondents with specific dietary motivations or health concerns were more likely to consult and engage with nutrition labels [21,23,35,38,40,44,45]. Rural adolescents faced difficulties in understanding labels presented in non-native languages such as English and struggled with unfamiliar nutrient-related terms and symbols [38]. Qualitative findings highlighted recurring barriers that limited engagement – such as brand reliance, limited time, small font sizes, technical jargon, poor awareness of nutrient functions, and the perception that label reading was unnecessary. While these factors influence consumers’ food label awareness, perceptions of label elements also play a pivotal role in shaping label engagement and comprehension. Table 1 summarises the determinants influencing food label literacy identified across the reviewed studies.

**Table 1.** Determinants of food label literacy mentioned by the reviewed articles.

Category	Determinants
Socio-demographic Characteristics	Gender, Age, Education level, Parental education, Marital status, Occupation, Income, Urban – rural residence, Presence of co-morbidities, Media exposure (presence of TV in bedroom), Family history of NCDs, Family composition (presence of pregnant women, young children)
Food Label/Nutrition Literacy	Awareness of nutrition labels, Knowledge of nutrient functions, Ability to interpret label terms (e.g. calories, trans fat, artificial sweeteners), Understanding of symbols or logos
Label Format and Design Factors	Font size, Language (e.g. English vs native), Overcrowding of information, Technical complexity (per 100 g vs per-serving values), Label location (BoP vs FoP), Differential perceptions of FoPL formats (e.g. MTL, HSR, Warning Label)
Behavioural and Psychosocial Factors	Time constraints, Brand trust or familiarity, Scepticism toward nutrition or health claims, Taste preferences, Media/celebrity/peer influence, Purchase habits, Health-related and psychosocial motivations (e.g. weight management, dietary conditions, specific conditions like diabetes or high cholesterol, and perceived alignment of health claims with body image ideals), Perceived usefulness of labels

Note: NCDs = Non-communicable diseases; FoP = Front-of-pack; BoP = Back-of-pack; FoPL = Front-of-pack label; MTL = Multiple traffic lights; HSR = Health star rating.

### Consumers’ food label perceptions: engagement, comprehension and design cues

Across studies, participants consistently recognised food labels as useful tools – especially for checking expiry dates, prices, brand names, and vegetarian symbols. However, engagement with nutritional details such as sodium, sugar, fat, or ingredient lists was often limited, selective, and superficial [39,43]. Despite high awareness of basic food label information among healthcare providers and medical students in India, engagement is primarily limited to checking expiry dates and price. The use of nutritional information to guide purchasing decisions remains low [32,39,46–48]. Even among participants who self-reported as label readers, familiarity with terms such as ‘artificial sweeteners’ was limited, and many struggled to interpret claims like ‘trans-fat free,’ revealing a persistent gap between food label recognition and functional comprehension [28,35,41,46].

The perceived utility of nutrition labels appeared to be constrained by cognitive and design-related barriers. Additional barriers, including technical terminology and low nutrition literacy, often intersected with format issues, such as overcrowded layouts or small font sizes. Engagement also varied depending on label type – particularly between back-of-pack (BoP) and FoPL – and across different consumer segments [23,29,48–50]. Still, interpretive FoPL formats were strongly preferred across experimental and observational studies over traditional BoP layouts. Formats such as traffic light labels, health star ratings (HSR), and warning labels were consistently rated as easier to interpret and more aligned with time-sensitive decisions at the point of purchase [20,24,25,29,48,50,51]. Experimental findings also showed that colour-coded and summary formats improved consumers’ ability to identify less healthy products and better understand nutrient risks, although these improvements did not consistently influence purchasing decisions [20,24].

Participants across several studies reported that critical nutritional details were too often relegated to the BoP, leading to calls for label repositioning and greater prominence of health information [29,48,51]. Consumer-driven suggestions to enhance label functionality included increasing font size, simplifying technical language, incorporating regional or local languages, using colour-coded summaries or pictograms, and deploying mass media campaigns to raise public awareness [26,27,37,38,48]. Adolescents recommended incorporating food label literacy into school curricula to build early awareness and comprehension [21,38]. Overall, participants viewed food labels as applicable – but underutilised – due to a mix of design flaws and low functional literacy.

### Food label engagement and its influence on purchase intentions

Across studies, a consistent theme was the disconnect between food label awareness, its comprehension, and translation into health-motivated purchase behaviour. Several studies found that participants either felt unaffected by label information or did not modify their purchase decisions based on it. When label use did influence intention, it often aligned with specific motivations – such as health claims related to body image ideals. Respondents frequently cited taste, price, peer influence, media exposure, and celebrity endorsements as stronger drivers of food choice than food labels [21,22,26,28,33,40–42,44,45,49]. Notably, most studies relied on self-reported behaviours, limiting the ability to assess real-world behavioural outcomes of food label use.

Only a small proportion of the general population reported consistent use of labels before purchase [34,35,48], and few believed that nutrition labels meaningfully shaped their decisions [26,31,46,47]. Even when nutritional information was noticed, it was not always interpreted correctly, weakening its behavioural influence [24,43]. One study identified a positive association between regular label reading and healthier food choices, particularly among those with specific dietary goals [36]. When motivation for label use existed, it was typically anchored in health concerns such as managing non-communicable diseases [23,27]. Simplified FoPLs, including HSR and warning labels, were more likely to influence purchase intentions than detailed BoP formats – suggesting that interpretive simplicity facilitates decision-making in practice [25]. Another study found only a weak positive correlation between label knowledge and attitude and practice, indicating that improved understanding alone may not guarantee behaviour change [48].

Experimental studies added nuance to these findings. One large multi-centre trial reported no significant change in purchase intentions following FoPL exposure, although perceived message effectiveness improved [20]. An eye-tracking study showed that formats such as Nutri-Score successfully captured visual attention and shaped product perception – but the authors cautioned that these patterns may not hold in real-world shopping contexts [51]. A discrepancy between experimental improvements in comprehension and minimal real-world behavioural change hinders the challenge of translating cognitive awareness into sustained consumer practices.

In summary, while people recognise food labels as informative tools, their behavioural impact remains modest. Convenience, brand familiarity, and marketing cues often override label use, underscoring the persistent intention – action gap in food label engagement.

## Discussion

In alignment with Sustainable Development Goal (SDG) 3, particularly target 3.4, which aims to reduce premature mortality from NCDs through prevention and health promotion, this scoping review maps the current evidence on consumer engagement with food labels – an important yet underutilised tool for enabling informed food choices [52]. This scoping review synthesised evidence from 32 primary studies across Indian states and institutional settings, reflecting disciplinary diversity and predominantly quantitative designs. Despite increased visibility of food labels in the Indian retail environment, most studies highlighted limited consumer engagement beyond basic components such as expiry date and price. Moreover, variations in socio-demographic factors, low functional literacy, and reliance on self-reported behaviours limited the understanding of how nutrition label components influence purchasing behaviour. Globally, researchers have reported similar evidence and methodological limitations, where awareness does not consistently lead to adequate label engagement [9,53]. These findings underscore the need for a deeper, context-sensitive understanding of consumer – label interactions. The review reflects on this through a thematic synthesis and considers how knowledge co-creation, mobilisation, and translation can inform future research addressing identified gaps.

### Determinants of food label literacy

Indian studies identified gender as the most consistent determinant of label use, with women showing greater engagement across label components. Other socio-demographic variables – such as age, income, education, and urban/rural residence – showed mixed associations, while health motivations, underlying health conditions, and family composition (e.g. presence of pregnant women, young children, or members with NCDs) were among the least explored. These findings are consistent with evidence from Global South contexts, such as China and Ecuador, which indicates that gender, education, income, cohabitation with pregnant women, and individual health motivations influence label engagement [10,54–56]. Studies of a scoping review from Arab countries identified nutrition literacy as the primary food label engagement determinant, with education, age, and income emerging as secondary predictors [9]. General and health and nutrition-specific literacy is linked to consumers’ ability to comprehend label content [8,56]. These findings underscore the significance of context-specific interventions that consider local socio-demographic profiles – alongside the broader behavioural, psychosocial, and commercial determinants identified in our review – to enhance label engagement and facilitate informed purchase decisions.

### Cognitive barriers and design constraints

Across the Indian evidence base, consumers reported awareness of nutrition terms or symbols but struggled with comprehension. Poor health literacy, technical jargon, non-native language presentation, and small or crowded label designs limited the utility. Globally, these barriers are echoed across diverse settings. Health literacy and label comprehension scoping review found that individuals with lower numeracy and literacy face significant difficulties comprehending nutrition labels, particularly BoP formats [8]. Experimental research from Italy confirmed that even when label formats are technically precise, consumers often struggle to interpret specific elements such as ingredient order, technical terminology, acronyms, and symbols [10]. Similarly, a study from China found that higher nutrition literacy was associated with more frequent use of labels – particularly for interpreting nutrition facts, nutrition claims, and stated health benefits [56].

Indian studies have reported the preferences for simplified FoPLs – such as Multiple Traffic Light (MTL), HSR, and warning labels aligning with global evidence highlighting interpretive formats’ effectiveness in aiding consumer understanding. A large-scale randomised trial from France found the Five-Colour Nutrition Label, followed by MTL, to effectively enhance the nutritional quality of consumer purchases. Notably, the effectiveness of these label formats did not vary significantly across sociodemographic characteristics – suggesting that simplified, interpretive, and colour-coded labels may have broad utility across diverse consumer segments [57]. Similarly, Kenya’s randomised trial showed that Black octagon warning labels significantly improved nutrient identification and reduced purchase intentions of unhealthy foods, underscoring the importance of simple, clear, and interpretable FoPL design [58]. In Chile, the Black-and-White stop sign design showed better comprehension. The design influenced purchase intentions, emphasising that clear and straightforward visual cues in label design can be processed quickly during real-world shopping [59].

Additionally, mass media campaigns to raise public awareness suggested by Indian consumers align with lessons from Chile, where a government-led communication initiative accompanying the FoPL rollout contributed to measurable reductions in the purchase of calories, sugar, saturated fat, and sodium. These effects observed across diverse socio-economic groups highlight the potential of nutrition – and food label literacy – to drive population-level dietary improvements [11,59]. Mirroring this approach, the advice to consumers given by the ICMR – NIN Dietary Guidelines for Indians (2024) is to scrutinise key nutrients, emphasising engagement with food labels as a critical step towards informed and healthier dietary choices [3].

A recent Indian study examining 230 packaged food products found that over 80% of on-pack claims were unverifiable due to vague regulatory definitions or the absence of quantitative disclosure. Several products also featured misleading claims despite being high in sugar, salt, or saturated fat [60]. Such inconsistencies in labelling practices and enforcement may contribute to consumer mistrust – an issue echoed in several studies reviewed here, where scepticism towards nutrition and health claims was frequently reported as a barrier to comprehension and use. The ICMR – NIN guidelines caution that loosely regulated health and nutrition claims may create a misleading ‘health halo,’ potentially confusing consumers [3]. These concerns reinforce the dual need to strengthen food label literacy – particularly around interpreting claims – and enhance regulatory oversight to support well-informed purchasing decisions.

### Food labels and consumer decision-making

A key finding from this review is the limited engagement and behavioural influence of food labels, with many consumers prioritising taste, price, brand familiarity, and social cues over nutritional information. A recent scoping review highlighted key methodological limitations in current research – most notably the overreliance on self-reported label use and conceptual overlap between health literacy and label comprehension. To address these gaps, objective behavioural methods such as eye-tracking technology were recommended to validate engagement and refine assessments of label comprehension [8]. Although Indian experimental studies showed some impact of FoPLs on attention and perception, these effects did not translate into consistent engagement. International evidence similarly reflects this disconnect. A systematic review found that the association between label use and dietary behaviour was inconsistent. However, label users often reported healthier dietary intake, when motivated by weight loss or disease prevention [53]. However, real-world impact remains modest, partly due to reliance on self-reported data, experimental conditions, and social desirability bias. While experimental formats may attract visual attention, their practical utility depends on whether consumers can comprehend and act on the information effectively while purchasing packaged food items. Hence, the review underscored the need for empirical research that evaluates how consumers interpret and apply ingredient lists, serving size information, and FoPLs in real-world purchase decisions [53]. Together, advancing the science of food label evaluation will require methodological innovation and a stronger emphasis on real-world decision-making environments.

A recent meta-analysis of 60 intervention studies identified limited attention, comprehension, and motivation as persistent consumer-level barriers to food label use. Despite these challenges, the analysis reported that food labelling improved consumer dietary behaviours – reducing energy intake, total fat, and other unhealthy food choices. Factors like label placement, intervention duration, and whether the labelling was voluntary or mandated did not influence the effectiveness of labelling interventions [61]. These findings echo Indian respondents’ preferences for simpler FoPLs, and highlight labelling as a potential intervention to influence consumer behaviour. To strengthen this impact, and in line with lessons from Chile, the meta-analysis recommended integrated strategies that combine food labelling with mass media campaigns, economic incentives, and supportive food environments [59,61]. These complementary approaches – including early-age interventions, reformulation incentives, and regulatory oversight – may help bridge the gap between label comprehension and healthier purchasing decisions.

### Knowledge co-creation for evidence

Grounded in the need for robust, context-sensitive evidence, this section highlights avenues for knowledge co-creation – emphasising methodological innovation and inclusive research strategies to map real-world behavioural insights that can inform policy and practice [62]. To advance current understanding, future studies must move beyond controlled experimental settings and adopt behavioural experiments, longitudinal tracking, and field-based designs in retail environments to capture how consumers engage with food labels in real-life contexts. Complementary approaches – such as cohort studies, eye-tracking technologies, crowd-sourced data, and analysis of retail sales data – could provide insights into food label engagement and purchasing behaviour [8,11,51,57,63]. Eye-tracking studies in retail settings could capture consumers’ visual attention to specific label components, duration, and sequence. The correlation of actual sales with this behavioural data can provide powerful insights into the real-world impact of labelling policies. This approach shows a direct link between consumers’ visual engagement with food labels and their purchasing decisions, which may be more reliable than relying on self-reported intentions.

Future studies need to examine how food label engagement varies across product categories – such as beverages, snacks, dairy, and convenience foods – because evidence suggests that it influences consumer attention and response to labels [24,38]. A randomised trial in Canada found that interpretive FoPL had differential effects across categories, with formats like HSR encouraging purchases of perceived healthier options, while ‘High In’ warnings more effectively discouraged selection of less healthy items like chocolate milk [64]. These insights can inform the development of category-specific labelling strategies and public health interventions by accounting for the cognitive processes that consumers engage in during purchase decisions.

Consistent with global reviews, evidence remains limited on how consumers interpret and apply ingredient lists and serving size information, underscoring the need for more nuanced investigations that prioritise subgroups with low nutrition literacy – such as adolescents, rural populations, or individuals from low-income settings – who may derive the most benefit from simplified, interpretive formats [8,31,34,38,53,56]. Regulatory attention is also warranted to address inconsistencies in nutrition and health claims, particularly where promotional language conflicts with nutrient declarations on HFSS products [26,41,60]. Food industry stakeholders – manufacturers and retail chains – are central to advancing food label research. Their contributions, through Corporate Social Responsibility funding, label design expertise, product reformulation, and mass communication, can enhance consumer awareness and support shared public health objectives.

### Knowledge mobilisation for policy to practice

Grounded in the evidence base, this section outlines actionable pathways for knowledge mobilisation – emphasising stakeholder engagement, and the integration of food label literacy into national education policy and public health programmes [65–67]. For instance, adolescents and young adults underscored the critical need for food label literacy – a direct reinforcement of the NEP 2020s vision to equip learners at all stages with competencies to address real-world challenges [16]. The National Council of Educational Research and Training has integrated concepts of hygiene and nutrition into its Environmental Studies curriculum, particularly for primary school, providing an immediate entry point for curricular alignment [68]. Activities focused on nutrition awareness could explicitly incorporate food label literacy. Such initiatives could empower children to become agents of change, influencing family members label engagement and subsequent purchase intentions [69]. For young adults, particularly undergraduate students, India’s Massive Open Online Courses platform – SWAYAM, could consider offering structured modules in food label literacy, either as part of mandatory life skills education or as electives within existing degree curricula. These modules could be linked to the Academic Bank of Credits to provide formal academic recognition [16,70]. Such integration would simultaneously advance NEP’s goals of application-oriented learning methods to develop skills, practical learning approaches in nutritional education, and basic training in health, including nutrition. For broader public outreach, initiatives like the Fit India Movement, which spans schools, colleges, workspaces, and community settings, can be leveraged to integrate food label literacy meaningfully into ongoing efforts emphasising nutrition awareness and promoting healthy eating behaviours [71].

Mobilising knowledge into practice requires the active engagement of regulatory bodies (e.g. FSSAI), policymakers, nutrition and behavioural scientists, educators, media, and public health professionals. Recognising the interdependence of food systems, public health, education, and governance, a whole-of-systems approach offers a strategic path forward [72]. Equitable label design, informed by cross-sectoral collaboration, can enable responsive implementation and strengthen public trust. Examples from Global South countries, including Mexico and Chile, demonstrated how participatory, evidence-informed policymaking can shape effective labelling reforms [59,73,74]. In the Indian context, national agencies such as ICMR – NIN and Anusandhan-National Research Foundation (A-NRF) could similarly catalyse a pan-India research and implementation agenda – curating standardised tools, coordinating research networks, and supporting real-time data analytics [75,76].

### Knowledge translation for accelerating research utilisation

To overcome persistent gaps in food label awareness, comprehension, and behavioural application, this section explores how technological innovations – grounded in precision nutrition and precision public health – can enable real-time, scalable, and user-centric applications. Translational efforts shall harness AI-powered tools, mobile applications, QR-based scanners, wearable technologies, and crowd-sourced databases to bridge the gap between label comprehension and informed decision-making at the point of purchase [63,77]. These innovations hold particular relevance in the Indian context, where expanding digital infrastructure, low data costs, and high smartphone penetration offer unique opportunities to personalise purchasing recommendations based on food labels, tailored to an individual’s or household’s sociodemographic and health profiles.

Despite increasing food label awareness, this review highlights persistent gaps in consumers’ ability to comprehend and apply label information, particularly regarding nutrient declarations, ingredient lists, and serving size details. These limitations are compounded by broader health and nutrition literacy challenges, as well as India’s population diversity and contextual complexities. Traditional awareness campaigns alone may be insufficient to influence purchasing decisions at scale. Emerging technologies offer promising pathways to navigate these barriers. For instance, smartphone-based image scanner applications could allow users to scan or photograph food labels, set up basic demographic or health-related profiles, enabling personalised assessments of food products at the point of sale [78–80]. Tailored recommendations generated by their algorithms can match label content to an individual’s or household’s dietary preferences, health conditions, and sociodemographic profiles. The lead in advancing digital solutions tailored to the Indian context can be taken by regulatory bodies such as FSSAI and national institutions like ICMR – NIN and A-NRF. This involves support for the comprehensive algorithm development that accounts for the country’s sociodemographic diversity, dietary patterns, and health burdens. Such tools could be promoted for public adoption through nationwide health promotion initiatives such as the Fit India Movement. Alternatively, such tools could be embedded within retail ecosystems where supermarkets and e-commerce platforms deploy them to enhance customer experience and loyalty. Although industry involvement in food label research remains limited, shifts in consumer demand driven by these tools may incentivise manufacturers to respond through product reformulation and improved label clarity.

Future innovations may also include wearable technologies (e.g. heads-up displays and smart glasses) capable of tracking visual attention to labels, supporting on-the-spot interpretation, and offering real-time purchasing recommendations [81]. These technologies not only enhance consumer comprehension but also generate granular behavioural data – capturing how labels are read, understood, and acted upon – which can, in turn, inform continuous optimisation of labelling strategies and contribute to the broader evidence base. By applying frameworks from knowledge translation research – which emphasises bridging evidence to policy and practice through stakeholder-driven, multimodal strategies this vision furthers a science-informed, digitally-enabled approach to optimising food label effectiveness [82].

**Figure 3.**
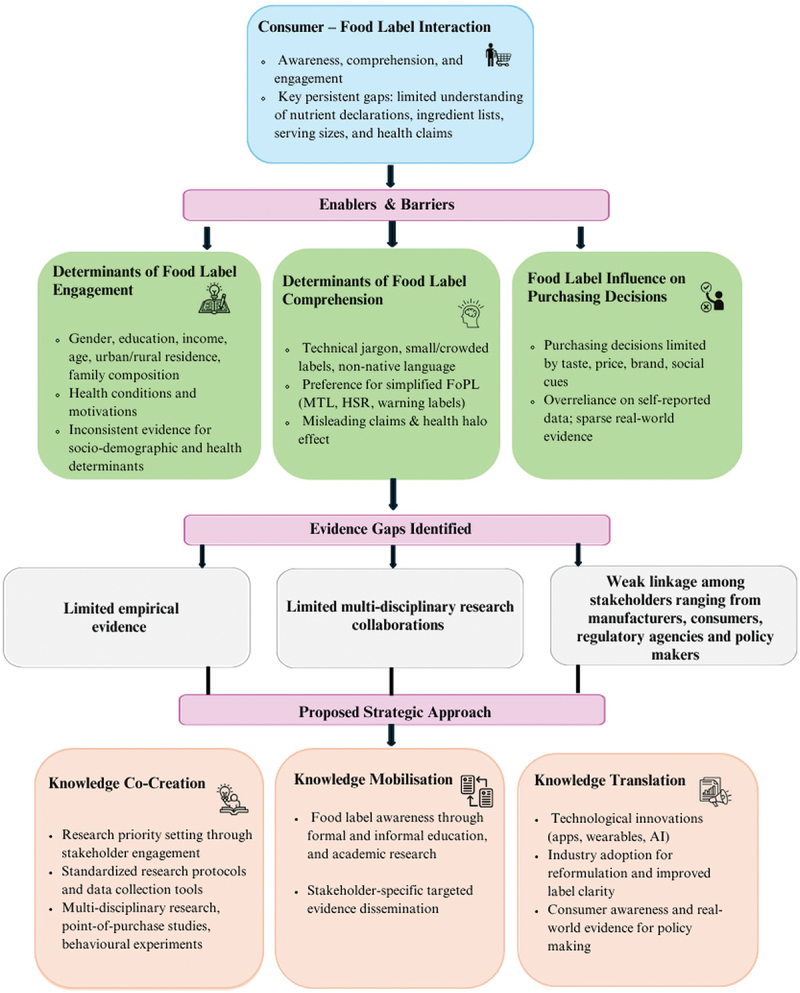
Framework summarising evidence and strategies for food label engagement, comprehension and purchase decisions.

To summarise the synthesis and guide future research, we present a conceptual framework linking key findings and evidence gaps across the three themes (Figure 3). The framework also outlines strategies to address these gaps and enhance consumer engagement through knowledge co-creation, mobilisation, and translation approaches.

### Strengths and limitations of the review

Strengths of this review include its decade-long coverage, inclusion of studies from multiple disciplines, and the integration of literature from conventional databases and Google Scholar, enabling the inclusion of India-specific research. However, most studies relied on self-reported data, with limited use of experimental or observational methods to capture actual food label engagement or purchasing behaviour. This limitation reflects global concerns, particularly the overreliance on cross-sectional data and the limited use of technologies such as eye-tracking or digital purchase audits to validate self-reported label use [11,56].

## Conclusion

Food labels remain a promising yet underutilised tool to support healthier packaged food choices. Although awareness of basic food label elements is relatively high, this review reveals persistent gaps in nutrition information comprehension and in behavioural engagement with labels during purchase decisions. These challenges stem from design complexity, limited health and nutrition literacy, and dominant market influences. These insights are timely as India navigates a nutrition transition marked by rising consumption of ultra-processed foods. Notably, several included studies mirror the formative research efforts that guided FoPL policies in countries like Chile, Kenya, and Mexico – underscoring India’s potential to contribute regionally and across the Global South. To bridge the intention – action gap in food label use, we need to simplify FoPL formats, use evidence-based education, and foster multi-sectoral collaboration.

To advance policy and practice, future efforts must adopt a systems lens – reviewing existing policies across the food regulation, health promotion, and education sectors to embed nutrition literacy, including food label awareness, within broader public health strategies. Technological innovations, such as QR code – enabled apps and personalised decision-support tools, offer scalable avenues to strengthen consumer engagement and data-driven implementation. Ultimately, improving food label literacy can support healthier food systems and contribute to SDG 3.4 (reducing premature mortality from NCDs), SDG 4.7 (education for sustainable development and health literacy), SDG 12.8 (ensuring consumers have information for sustainable lifestyles), and SDG 17.16 (enhancing the global, multi-stakeholder partnerships).

## Supplementary Material

PRISMA_Checklist.docx

Clean copy _Supplementary_File.docx

## Data Availability

CRediT: Conceptualisation: PN, MP, KT; Data curation: MP, KT; Formal analysis: MP, KT; Methodology: PN, MP, KT; Supervision: PN, SL; Writing – original draft: KT, MP; Writing – review & editing: PN, SL.

## Data availability statement

The data synthesised in this review consist of previously published studies, all of which are cited within the manuscript. Additional information regarding the data extraction and charting process is available from the corresponding author upon reasonable request.
