# Supplementary material for: Food labelling in India: a scoping review of consumer engagement, comprehension, and purchase behaviour
Source: Glob Health Action. 2025 Nov 11;18(1):2574132. doi: 10.1080/16549716.2025.2574132 (PMC12608109; doi:10.1080/16549716.2025.2574132)
Supplement: Clean copy _Supplementary_File.docx [file ZGHA_A_2574132_SM8663.docx]

**Supplementary Tables**

**Table S1: Search strategy used in the screening process**

| **Database (Search Date)** | | **Search string** | |
| --- | --- | --- | --- |
| PubMed  (17 February 2025) | | (("food labeling"[MeSH Terms] OR "food label*"[Text Word] OR "nutrition* label*"[Text Word] OR "food label use"[Text Word]) AND ("choice behavior"[MeSH Terms] OR "choice behavior"[Text Word] OR "consumer behavior"[MeSH Terms] OR "food preferences"[MeSH Terms] OR "food preference"[Text Word] OR "purchasing behavior"[Text Word] OR "purchasing behaviour"[Text Word] OR "purchasing intention"[Text Word] OR "buying behavior"[Text Word] OR "buying haviour"[Text Word] OR "buying intention"[Text Word] OR "food choices"[Text Word] OR "Diet"[MeSH Terms] OR "Diet"[Text Word]) AND ("India"[MeSH Terms] OR "India"[Text Word] OR "Indian"[Text Word])) | |
| Web of Science  (20 February 2025) | | (("food labeling" OR "food label*" OR "nutrition* label*" OR "food label use") AND ("choice behavior" OR "choice behavior" OR "consumer behavior" OR "food preferences" OR "food preference" OR "purchasing behavior" OR "purchasing behaviour" OR "purchasing intention" OR "buying behavior" OR "buying behaviour" OR "buying intention" OR "food choices" OR Diet OR Diet) AND (India OR India OR Indian)) | |
| **Database (Search Date)** | **Search string** | |  |
| Scopus  (17 February 2025) | ( ( INDEXTERMS ( "food labeling" ) OR TITLE-ABS-KEY ( "food label*" ) OR TITLE-ABS-KEY ( "nutrition* label*" ) OR TITLE-ABS-KEY ( "food label use" ) ) AND ( INDEXTERMS ( "choice behavior" ) OR TITLE-ABS-KEY ( "choice behavior" ) OR INDEXTERMS ( "consumer behavior" ) OR INDEXTERMS ( "food preferences" ) OR TITLE-ABS-KEY ( "food preference" ) OR TITLE-ABS-KEY ( "purchasing behavior" ) OR TITLE-ABS-KEY ( "purchasing behaviour" ) OR TITLE-ABS-KEY ( "purchasing intention" ) OR TITLE-ABS-KEY ( "buying behavior" ) OR TITLE-ABS-KEY ( "buying behaviour" ) OR TITLE-ABS-KEY ( "buying intention" ) OR TITLE-ABS-KEY ( "food choices" ) OR INDEXTERMS ( diet ) OR TITLE-ABS-KEY ( diet ) ) AND ( INDEXTERMS ( india ) OR TITLE-ABS-KEY ( india ) OR TITLE-ABS-KEY ( indian ) ) ) | |  |
| CINAHL  (17 February 2025) | ((((MH "food labeling+") OR "food label*" OR "nutrition* label*" OR "food label use") AND ((MH "choice behavior+") OR "choice behavior" OR (MH "consumer behavior+") OR (MH "food preferences+") OR "food preference" OR "purchasing behavior" OR "purchasing behaviour" | |  |

| **Database (Search Date)** | **Search string** |
| --- | --- |
| CINAHL | OR "purchasing intention" OR "buying behavior" OR "buying behaviour" OR "buying intention" OR "food choices" OR (MH Diet+) OR Diet)) AND (((MH India+) OR India OR Indian))) |
| Embase  (17 February 2025) | ('food labeling'/exp OR 'food label*':ti,ab,kw,de,dn,df,mn,tn OR 'nutrition* label*':ti,ab,kw,de,dn,df,mn,tn OR 'food label use':ti,ab,kw,de,dn,df,mn,tn) AND ('decision making'/exp OR 'choice behavior':ti,ab,kw,de,dn,df,mn,tn OR 'consumer attitude'/exp OR 'food preference'/exp OR 'food preference':ti,ab,kw,de,dn,df,mn,tn OR 'purchasing behavior':ti,ab,kw,de,dn,df,mn,tn OR 'purchasing behaviour':ti,ab,kw,de,dn,df,mn,tn OR 'purchasing intention':ti,ab,kw,de,dn,df,mn,tn OR 'buying behavior':ti,ab,kw,de,dn,df,mn,tn OR 'buying behaviour':ti,ab,kw,de,dn,df,mn,tn OR 'buying intention':ti,ab,kw,de,dn,df,mn,tn OR 'food choices':ti,ab,kw,de,dn,df,mn,tn OR 'diet'/exp OR 'diet':ti,ab,kw,de,dn,df,mn,tn) AND ('india'/exp OR 'india':ti,ab,kw,de,dn,df,mn,tn OR 'indian':ti,ab,kw,de,dn,df,mn,tn) |

**Table S2: Overview of included studies**

| **Author, year** | **Source/ Database** | **Journal** | **Inter-disciplinary Collaboration** | **Departments Involved** | **Study State(s)** | **Methodological Approach** | **Sample Size** | **Participants** |
| --- | --- | --- | --- | --- | --- | --- | --- | --- |
| Kansal et al. 2022 | PubMed | Appetite | Yes | Department of Community Medicine, Department of Sociology, School of Exercise and Nutrition Sciences | Uttar Pradesh | QUAL | 44 | adolescents |
| Singh et al. 2022 | PubMed | Nutrients | Yes | Department of Survey Research and Data Analytics, Department of Nutrition, Carolina Population Center, Global Public Health NGO | Gujarat, Odisha, Delhi, Assam, Uttar Pradesh, Karnataka | QUAN | 2869 | adults |
| Sindhu & Madaiah, 2023 | PubMed | Journal of Family Medicine and Primary Care | No | Department of Community Medicine | Karnataka | QUAN | 200 | adolescents |
| Bhattacharya et al. 2022 | PubMed | Frontiers in Public Health | Yes | Global Health Advocacy NGO, Department of Community Medicine | Fourteen States | QUAN | 2024 | adults |
| M. Verma et al. 2023 | PubMed | BMC Public Health | No | Department of Community Medicine | Punjab | QUAN | 722 | adolescents-parents duo |
| Saha et al. 2021 | Scopus | Journal of Content, Community & Communication | Yes | Nutrition Information Communication and Health Education, Food & Drug toxicology Research Centre, Department of Food & Nutrition | Telangana | QUAN | 350 | adolescents |
| Srivastav et al. 2022 | Scopus | Food Research | Yes | Department of Nutrition and Dietetics, Department of Home Science | Uttar Pradesh | QUAN | 100 | adults (women) |
| Choudhary et al.2024 | Scopus | Springer Proceedings in Business and Economics | No | Marketing | Maharashtra | QUAN | 70 | young adults |
| Srishti Mediratta & Mathur, 2023 | Web of Science | Health Education Journal | No | Department of Food and Nutrition and Food Technology | New Delhi | QUAN | 589 | adults |
| Pettigrew et al. 2023 | Web of Science | Food Quality and Preference | Yes | Food Policy, Centre for Behaviour Change, Non-Academic Research | Not mentioned | Mixed Methods | 1270; 112 | adults |

| **Author, year** | **Source/ Database** | **Journal** | **Inter-disciplinary Collaboration** | **Departments Involved** | **Study State(s)** | **Methodological Approach** | **Sample Size** | **Participants** |
| --- | --- | --- | --- | --- | --- | --- | --- | --- |
|  |  |  |  | Institute, School of Public Health, Global Health NGO, Multilateral Organizations |  |  |  |  |
| Kumar & Kapoor, 2017 | Google Scholar | British Food Journal | Yes | Rural Management, Centre of Food and Agri-Business Management | Not mentioned | QUAN | 300 | young adults |
| Ghosh et al. 2023 | Web of Science | Food Quality | Yes | Centre for Management | Andhra Pradesh, | QUAN | 20,564 | adults |
| **Author, year** | **Source/ Database** | **Journal** | **Inter-disciplinary Collaboration** | **Departments Involved** | **Study State(s)** | **Methodological Approach** | **Sample Size** | **Participants** |
|  |  | and Preference |  | in Agriculture, Marketing | Assam, Bihar, Chhattisgarh, Delhi, Gujarat, Haryana, Himachal Pradesh, Jharkhand, Karnataka, Kerala, Madhya Pradesh, Maharashtra, |  |  |  |

| **Author, year** | **Source/ Database** | **Journal** | **Inter-disciplinary Collaboration** | **Departments Involved** | **Study State(s)** | **Methodological Approach** | **Sample Size** | **Participants** |
| --- | --- | --- | --- | --- | --- | --- | --- | --- |
|  |  |  |  |  | NE Group (Arunachal Pradesh, Meghalaya, Mizoram, Manipur, Tripura, and Nagaland), Odisha, Punjab, Rajasthan, Tamil Nadu, Uttar Pradesh, West Bengal |  |  |  |

| **Author, year** | **Source/ Database** | **Journal** | **Inter-disciplinary Collaboration** | **Departments Involved** | **Study State(s)** | **Methodological Approach** | **Sample Size** | **Participants** |
| --- | --- | --- | --- | --- | --- | --- | --- | --- |
| P. Verma et al. 2024 | Web of Science | Indian Journal of Community Medicine | No | Department of Community Medicine | Maharashtra | QUAN | 226 | adolescents, adults |
| Suresh et al. 2024 | CINAHL | Indian Journal of Community Health | Yes | Department of Community Medicine, Center for Molecular Medicine and Therapeutics | Tamil Nadu | QUAN | 487 | adolescents, young adults |

| **Author, year** | **Source/ Database** | **Journal** | **Inter-disciplinary Collaboration** | **Departments Involved** | **Study State(s)** | **Methodological Approach** | **Sample Size** | **Participants** |
| --- | --- | --- | --- | --- | --- | --- | --- | --- |
| Patil et al. 2024 | CINAHL | Journal of the Scientific Society | No | Department of Community Medicine | Karnataka | QUAN | 206 | adolescents |
| Kathane & Sharma, 2017 | Google Scholar | IOSR Journal of Humanities and Social Science | Yes | Human Resource Development (Faculty Training), Department of Home Science | Maharashtra | QUAN | 200 | adults (women) |

| **Author, year** | **Source/ Database** | **Journal** | **Inter-disciplinary Collaboration** | **Departments Involved** | **Study State(s)** | **Methodological Approach** | **Sample Size** | **Participants** |
| --- | --- | --- | --- | --- | --- | --- | --- | --- |
| Kar et al. 2018 | Google Scholar | International Journal of Advanced Medical and Health Research | No | Department of Community Medicine | Pondicherry | QUAN | 153 | adults |
| Samit Dutta & Deval Patel, 2017 | Google Scholar | International Journal of Indian Psychology | No | Department of Food Business Management | Gujarat | QUAN | 150 | adults |

| **Author, year** | **Source/ Database** | **Journal** | **Inter-disciplinary Collaboration** | **Departments Involved** | **Study State(s)** | **Methodological Approach** | **Sample Size** | **Participants** |
| --- | --- | --- | --- | --- | --- | --- | --- | --- |
| Gopichandran & Annamalai, 2022 | Google Scholar | Indian Journal of Community and Family Medicine | No | Department of Community Medicine | Tamil Nadu | QUAN | 200 | young adults |
| Kamboj et al. 2022 | Google Scholar | The Indian Journal of Home Science | No | Department of Food and Nutrition | Delhi | QUAN | 570 | adolescents |

| **Author, year** | **Source/ Database** | **Journal** | **Inter-disciplinary Collaboration** | **Departments Involved** | **Study State(s)** | **Methodological Approach** | **Sample Size** | **Participants** |
| --- | --- | --- | --- | --- | --- | --- | --- | --- |
| Chellamuthu et al. 2024 | Google Scholar | Indian Journal of Community Medicine | No | Department of Community Medicine | Pondicherry | QUAN | 460 | adults |
| Kataria & Kouser, 2023 | Google Scholar | Global Journal of Medicine & Public Health | No | Department of Community Medicine | Jammu and Kashmir | QUAN | 228 | young adults |

| **Author, year** | **Source/ Database** | **Journal** | **Inter-disciplinary Collaboration** | **Departments Involved** | **Study State(s)** | **Methodological Approach** | **Sample Size** | **Participants** |
| --- | --- | --- | --- | --- | --- | --- | --- | --- |
| Diwakar et al. 2019 | Google Scholar | Journal of Pierre Fauchard Academy (India Section) | No | Department of Conservative Dentistry and Endodontics | Tamil Nadu | QUAN | 370 | adults |
| Selvamurthy et al. 2024 | Google Scholar | Journal of the Scientific Society | No | Department of Community Medicine | Tamil Nadu | Mixed Methods | 113; 8 | young adults |

| **Author, year** | **Source/ Database** | **Journal** | **Inter-disciplinary Collaboration** | **Departments Involved** | **Study State(s)** | **Methodological Approach** | **Sample Size** | **Participants** |
| --- | --- | --- | --- | --- | --- | --- | --- | --- |
| Patil et al. 2024 | Google Scholar | Journal of Dr. YSR University of Health Sciences | No | Department of Community Medicine | Not Mentioned | QUAN | 364 | adults |
| Sheikh, Bilkis A.;Patel, B.G, 2023 | Google Scholar | Journal of Progressive Agriculture | No | Department of Food Science and Nutrition | Gujarat | QUAN | 1000 | adults |

| **Author, year** | **Source/ Database** | **Journal** | **Inter-disciplinary Collaboration** | **Departments Involved** | **Study State(s)** | **Methodological Approach** | **Sample Size** | **Participants** |
| --- | --- | --- | --- | --- | --- | --- | --- | --- |
| Naik et al. 2024 | Google Scholar | Preventive Medicine Research & Reviews | No | Department of Community Medicine | Bihar | QUAN | 506 | adults |
| Gehlot & Soni, 2024 | Google Scholar | International Journal of Nutrition & Lifestyle | No | Department of Life Science | Gujarat, Rajasthan, Maharashtra | QUAN | 211 | adolescents, adults |

| **Author, year** | **Source/ Database** | **Journal** | **Inter-disciplinary Collaboration** | **Departments Involved** | **Study State(s)** | **Methodological Approach** | **Sample Size** | **Participants** |
| --- | --- | --- | --- | --- | --- | --- | --- | --- |
| Vadlamani et al. 2021 | Google Scholar | International Journal of Health and Clinical Research | No | Department of Community Medicine | Andhra Pradesh | QUAN | 127 | adults |
| Jadapalli & Somavarapu, 2018 | Google scholar | American Journal of Food Science and Nutrition | No | Department of Food Technology | Andhra Pradesh | QUAN | 100 | Not mentioned |

| **Author, year** | **Source/ Database** | **Journal** | **Inter-disciplinary Collaboration** | **Departments Involved** | **Study State(s)** | **Methodological Approach** | **Sample Size** | **Participants** |
| --- | --- | --- | --- | --- | --- | --- | --- | --- |
| Shamim et al. 2020 | Google scholar | Journal of Public Affairs | Yes | Business management, Department of Agricultural Economics and Business Management | Delhi | QUAN | 303 | adults |
| Singh et al. 2024 | Google Scholar | Preventive Medicine Research & Reviews | No | Department of Community Medicine | Rajasthan | QUAN | 100 | adults |
